# (2*E*)-3-(4-Fluoro­phen­yl)-1-[5-methyl-1-(4-methyl­phen­yl)-1*H*-1,2,3-triazol-4-yl]prop-2-en-1-one^1^

**DOI:** 10.1107/S1600536813008246

**Published:** 2013-04-05

**Authors:** Bakr F. Abdel-Wahab, Hanan A. Mohamed, Seik Weng Ng, Edward R. T. Tiekink

**Affiliations:** a Applied Organic Chemistry Department, National Research Centre, Dokki, 12622 Giza, Egypt; b Department of Chemistry, University of Malaya, 50603 Kuala Lumpur, Malaysia; c Chemistry Department, Faculty of Science, King Abdulaziz University, PO Box 80203 Jeddah, Saudi Arabia

## Abstract

With respect to the triazole ring in the title compound, C_19_H_16_FN_3_O, the *p*-tolyl ring is inclined [dihedral angle = 51.79 (11)°], whereas the chalcone residue is almost coplanar [O—C—C—N and C—C—C—C torsion angles = −178.71 (19) and 178.42 (18)°, respectively]. The conformation about the C=C bond [1.328 (3) Å] is *E*, and the triazole methyl group and the carbonyl O atom are *syn*. In the crystal, centrosymmetrically related mol­ecules are connected by π–π inter­actions between the triazole and *p*-tolyl rings [centroid–centroid distance = 3.6599 (12) Å] and these are linked into a three-dimensional architecture by C—H⋯N and C—H⋯π inter­actions.

## Related literature

For the biological activities of chalcone derivatives, see: Abdel-Wahab *et al.* (2012 ▶); Singh *et al.* (2012 ▶). For a related structure, see: Abdel-Wahab *et al.* (2013 ▶).
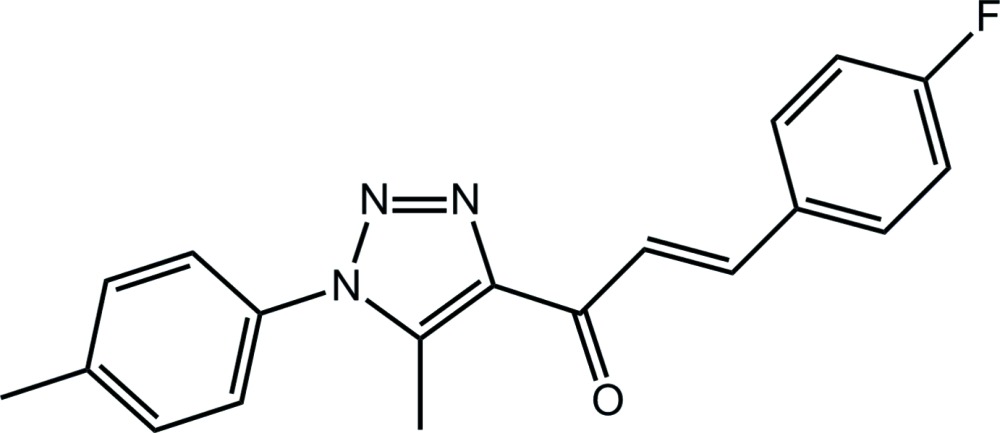


## Experimental

### 

#### Crystal data

C_19_H_16_FN_3_O
*M* *_r_* = 321.35
Triclinic, 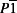

*a* = 6.2890 (5) Å
*b* = 10.8874 (8) Å
*c* = 11.9691 (9) Å
α = 101.144 (7)°
β = 92.634 (6)°
γ = 91.634 (6)°
*V* = 802.65 (11) Å^3^
*Z* = 2
Mo *K*α radiation
μ = 0.09 mm^−1^
*T* = 295 K
0.35 × 0.35 × 0.35 mm

#### Data collection

Agilent SuperNova Dual diffractometer with an Atlas detector
Absorption correction: multi-scan (*CrysAlis PRO*; Agilent, 2011 ▶) *T*_min_ = 0.887, *T*_max_ = 1.000
6854 measured reflections
3697 independent reflections
2308 reflections with *I* > 2σ(*I*)
*R*_int_ = 0.027

#### Refinement

*R*[*F* ^2^ > 2σ(*F* ^2^)] = 0.053
*wR*(*F* ^2^) = 0.139
*S* = 1.03
3697 reflections
220 parameters
H-atom parameters constrained
Δρ_max_ = 0.18 e Å^−3^
Δρ_min_ = −0.16 e Å^−3^

### 

Data collection: *CrysAlis PRO* (Agilent, 2011 ▶); cell refinement: *CrysAlis PRO*; data reduction: *CrysAlis PRO*; program(s) used to solve structure: *SHELXS97* (Sheldrick, 2008 ▶); program(s) used to refine structure: *SHELXL97* (Sheldrick, 2008 ▶); molecular graphics: *ORTEP-3 for Windows* (Farrugia, 2012 ▶) and *DIAMOND* (Brandenburg, 2006 ▶); software used to prepare material for publication: *publCIF* (Westrip, 2010 ▶).

## Supplementary Material

Crystal structure: contains datablock(s) global, I. DOI: 10.1107/S1600536813008246/hb7061sup1.cif

Structure factors: contains datablock(s) I. DOI: 10.1107/S1600536813008246/hb7061Isup2.hkl

Supplementary material file. DOI: 10.1107/S1600536813008246/hb7061Isup3.cml

Additional supplementary materials:  crystallographic information; 3D view; checkCIF report

## Figures and Tables

**Table 1:** Hydrogen-bond geometry (Å, °) *Cg*1 is the centroid of the C13–C18 benzene

*D*—H⋯*A*	*D*—H	H⋯*A*	*D*⋯*A*	*D*—H⋯*A*
C12—H12*C*⋯N3^i^	0.96	2.49	3.399 (3)	158
C2—H2⋯*Cg*1^ii^	0.93	2.91	3.650 (2)	138

Symmetry codes: (i) 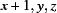; (ii) 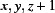.

**Fig. 1.**
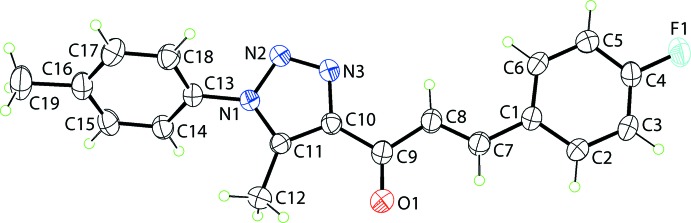
The molecular structure of (I) showing displacement ellipsoids at the 35% probability level.

**Fig. 2.**
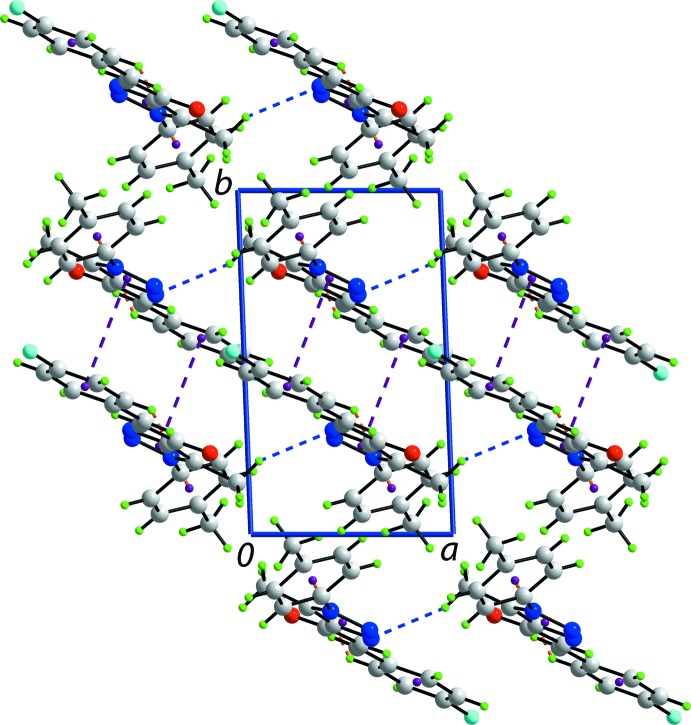
A view of the crystal packing in projection down the c axis. The C—H···N, C—H···π and π—π interactions are shown as blue, orange and purple dashed lines, respectively.

## supplementary crystallographic information

### Comment

Chalcone derivatives exhibit a range of biological activities (Abdel-Wahab *et al.*, 2012; Singh *et al.*, 2012) and in this connection, the title compound was synthesized and characterized crystallographically.

In (I), the *p*-tolyl ring attached to the triazole ring is inclined, forming a dihedral angle of 51.79 (11)°. By contrast, the chalcone residue is co-planar as seen in the values of the O1—C9—C10—N3 and C7—C8—C9—C10 torsion angles of -178.71 (19) and 178.42 (18)°, respectively. This co-planarity extends to include the terminal fluorobenzene ring [C6—C1—C7—C8 = 4.2 (3)°]. The conformation about the C7═C8 bond [1.328 (3) Å] is *E*, and the triazole-methyl and carbonyl-O1 substituents are *syn*. The conformation with respect to the triazole ring and chalcone residue resembles that found in a related compound (Abdel-Wahab *et al.*, 2013).

In the crystal structure, centrosymmetrically related molecules are connected by π—π interactions between the triazole and *p*-tolyl rings [inter-centroid distance = 3.6599 (12) Å, angle of inclination = 2.30 (11)° for symmetry operation: 1 - *x*, 1 - *y*, 2 - *z*]. The dimeric aggregates are connected into a three-dimensional architecture by C—H···N and C—H···π interactions, Fig. 2 and Table 1.

### Experimental

The title compound was prepared following the reported method (Abdel-Wahab *et al.*, 2012). Colourless blocks were obtained from its DMF solution by slow evaporation at room temperature.

### Refinement

Carbon-bound H-atoms were placed in calculated positions (C—H = 0.93 to 0.96 Å) and were included in the refinement in the riding model approximation, with *U*_iso_(H) = 1.2–1.5*U*_equiv_(C).

### Crystal data

**Table:**

C_19_H_16_FN_3_O	*Z* = 2
*M**_r_* = 321.35	*F*(000) = 336
Triclinic, *P*1	*D*_x_ = 1.330 Mg m^−^^3^
Hall symbol: -P 1	Mo *K*α radiation, λ = 0.71073 Å
*a* = 6.2890 (5) Å	Cell parameters from 1657 reflections
*b* = 10.8874 (8) Å	θ = 3.2–27.5°
*c* = 11.9691 (9) Å	µ = 0.09 mm^−^^1^
α = 101.144 (7)°	*T* = 295 K
β = 92.634 (6)°	Block, colourless
γ = 91.634 (6)°	0.35 × 0.35 × 0.35 mm
*V* = 802.65 (11) Å^3^	

### Data collection

**Table:**

Agilent SuperNova Dual diffractometer with an Atlas detector	3697 independent reflections
Radiation source: SuperNova (Mo) X-ray Source	2308 reflections with *I* > 2σ(*I*)
Mirror monochromator	*R*_int_ = 0.027
Detector resolution: 10.4041 pixels mm^-1^	θ_max_ = 27.6°, θ_min_ = 3.3°
ω scan	*h* = −8→6
Absorption correction: multi-scan (*CrysAlis PRO*; Agilent, 2011)	*k* = −12→14
*T*_min_ = 0.887, *T*_max_ = 1.000	*l* = −15→15
6854 measured reflections	

### Refinement

**Table:**

Refinement on *F*^2^	Secondary atom site location: difference Fourier map
Least-squares matrix: full	Hydrogen site location: inferred from neighbouring sites
*R*[*F*^2^ > 2σ(*F*^2^)] = 0.053	H-atom parameters constrained
*wR*(*F*^2^) = 0.139	*w* = 1/[σ^2^(*F*_o_^2^) + (0.0416*P*)^2^ + 0.2071*P*] where *P* = (*F*_o_^2^ + 2*F*_c_^2^)/3
*S* = 1.03	(Δ/σ)_max_ = 0.001
3697 reflections	Δρ_max_ = 0.18 e Å^−^^3^
220 parameters	Δρ_min_ = −0.16 e Å^−^^3^
0 restraints	Extinction correction: *SHELXL97* (Sheldrick, 2008), Fc^*^=kFc[1+0.001xFc^2^λ^3^/sin(2θ)]^-1/4^
Primary atom site location: structure-invariant direct methods	Extinction coefficient: 0.015 (2)

### Special details

**Table:**

Geometry. All s.u.'s (except the s.u. in the dihedral angle between two l.s. planes) are estimated using the full covariance matrix. The cell s.u.'s are taken into account individually in the estimation of s.u.'s in distances, angles and torsion angles; correlations between s.u.'s in cell parameters are only used when they are defined by crystal symmetry. An approximate (isotropic) treatment of cell s.u.'s is used for estimating s.u.'s involving l.s. planes.
Refinement. Refinement of *F*^2^ against ALL reflections. The weighted *R*-factor *wR* and goodness of fit *S* are based on *F*^2^, conventional *R*-factors *R* are based on *F*, with *F* set to zero for negative *F*^2^. The threshold expression of *F*^2^ > 2σ(*F*^2^) is used only for calculating *R*-factors(gt) *etc*. and is not relevant to the choice of reflections for refinement. *R*-factors based on *F*^2^ are statistically about twice as large as those based on *F*, and *R*- factors based on ALL data will be even larger.

### Fractional atomic coordinates and isotropic or equivalent isotropic displacement parameters (Å^2^)

**Table:**

	*x*	*y*	*z*	*U*_iso_*/*U*_eq_	
F1	−0.0563 (2)	0.53130 (13)	1.39178 (11)	0.0761 (4)	
N1	0.6205 (2)	0.22322 (14)	0.56828 (13)	0.0432 (4)	
N2	0.4313 (3)	0.27912 (17)	0.59390 (14)	0.0540 (5)	
N3	0.4258 (3)	0.30257 (16)	0.70426 (14)	0.0520 (4)	
O1	0.8127 (3)	0.23574 (16)	0.91088 (12)	0.0704 (5)	
C1	0.3444 (3)	0.37835 (17)	1.14542 (15)	0.0457 (5)	
C2	0.4017 (3)	0.38896 (19)	1.26083 (16)	0.0498 (5)	
H2	0.5333	0.3613	1.2821	0.060*	
C3	0.2675 (4)	0.43951 (19)	1.34448 (16)	0.0529 (5)	
H3	0.3067	0.4456	1.4213	0.064*	
C4	0.0766 (4)	0.48017 (19)	1.31138 (17)	0.0515 (5)	
C5	0.0113 (4)	0.4712 (2)	1.19918 (17)	0.0558 (6)	
H5	−0.1206	0.4995	1.1793	0.067*	
C6	0.1452 (3)	0.4193 (2)	1.11674 (17)	0.0533 (5)	
H6	0.1018	0.4114	1.0403	0.064*	
C7	0.4949 (3)	0.32849 (18)	1.06035 (17)	0.0501 (5)	
H7	0.6196	0.2985	1.0881	0.060*	
C8	0.4757 (4)	0.32071 (19)	0.94822 (16)	0.0521 (5)	
H8	0.3522	0.3474	0.9161	0.063*	
C9	0.6455 (3)	0.27077 (18)	0.87383 (16)	0.0483 (5)	
C10	0.6071 (3)	0.26277 (17)	0.75028 (16)	0.0429 (5)	
C11	0.7336 (3)	0.21146 (17)	0.66377 (15)	0.0415 (4)	
C12	0.9405 (3)	0.1506 (2)	0.66496 (19)	0.0604 (6)	
H12A	0.9507	0.0908	0.5952	0.091*	
H12B	0.9511	0.1085	0.7284	0.091*	
H12C	1.0540	0.2128	0.6721	0.091*	
C13	0.6637 (3)	0.17915 (17)	0.45098 (16)	0.0441 (5)	
C14	0.8548 (3)	0.21016 (19)	0.40945 (17)	0.0512 (5)	
H14	0.9561	0.2620	0.4565	0.061*	
C15	0.8934 (4)	0.1630 (2)	0.29706 (18)	0.0574 (6)	
H15	1.0225	0.1835	0.2690	0.069*	
C16	0.7455 (4)	0.08618 (19)	0.22491 (17)	0.0562 (6)	
C17	0.5531 (4)	0.0601 (2)	0.26836 (18)	0.0601 (6)	
H17	0.4492	0.0111	0.2207	0.072*	
C18	0.5117 (4)	0.10497 (19)	0.38069 (17)	0.0543 (5)	
H18	0.3822	0.0853	0.4087	0.065*	
C19	0.7960 (5)	0.0318 (2)	0.10330 (19)	0.0821 (8)	
H19A	0.6658	0.0068	0.0585	0.123*	
H19B	0.8820	−0.0399	0.1022	0.123*	
H19C	0.8722	0.0938	0.0720	0.123*	

### Atomic displacement parameters (Å^2^)

**Table:**

	*U*^11^	*U*^22^	*U*^33^	*U*^12^	*U*^13^	*U*^23^
F1	0.0828 (10)	0.0924 (10)	0.0550 (8)	0.0284 (8)	0.0226 (7)	0.0114 (7)
N1	0.0393 (9)	0.0511 (9)	0.0388 (9)	0.0029 (7)	0.0042 (7)	0.0068 (7)
N2	0.0441 (10)	0.0750 (12)	0.0431 (9)	0.0127 (9)	0.0055 (8)	0.0093 (9)
N3	0.0472 (10)	0.0665 (11)	0.0428 (9)	0.0104 (8)	0.0077 (8)	0.0095 (8)
O1	0.0638 (11)	0.1007 (13)	0.0487 (9)	0.0244 (9)	0.0017 (8)	0.0170 (9)
C1	0.0545 (13)	0.0444 (11)	0.0382 (10)	0.0018 (9)	0.0039 (9)	0.0078 (9)
C2	0.0528 (13)	0.0565 (12)	0.0416 (11)	0.0052 (10)	−0.0002 (9)	0.0138 (9)
C3	0.0643 (15)	0.0604 (13)	0.0334 (10)	0.0001 (11)	0.0014 (10)	0.0080 (9)
C4	0.0610 (14)	0.0514 (12)	0.0432 (11)	0.0070 (10)	0.0123 (10)	0.0085 (9)
C5	0.0539 (14)	0.0654 (14)	0.0494 (12)	0.0118 (11)	0.0015 (10)	0.0135 (11)
C6	0.0592 (14)	0.0644 (13)	0.0356 (10)	0.0053 (11)	−0.0031 (10)	0.0090 (10)
C7	0.0556 (13)	0.0509 (11)	0.0444 (11)	0.0054 (10)	0.0038 (10)	0.0098 (9)
C8	0.0594 (14)	0.0567 (12)	0.0412 (11)	0.0093 (10)	0.0060 (10)	0.0099 (9)
C9	0.0554 (14)	0.0474 (11)	0.0418 (11)	0.0031 (10)	0.0043 (10)	0.0076 (9)
C10	0.0426 (11)	0.0441 (10)	0.0415 (10)	−0.0001 (8)	0.0038 (9)	0.0074 (9)
C11	0.0404 (11)	0.0436 (10)	0.0410 (10)	−0.0005 (8)	0.0018 (9)	0.0101 (8)
C12	0.0488 (13)	0.0772 (15)	0.0562 (13)	0.0137 (11)	0.0039 (10)	0.0139 (12)
C13	0.0495 (12)	0.0443 (10)	0.0382 (10)	0.0024 (9)	0.0065 (9)	0.0065 (8)
C14	0.0504 (13)	0.0537 (12)	0.0479 (11)	−0.0015 (10)	0.0074 (10)	0.0053 (10)
C15	0.0613 (15)	0.0612 (13)	0.0529 (13)	0.0061 (11)	0.0198 (11)	0.0145 (11)
C16	0.0801 (17)	0.0473 (12)	0.0438 (11)	0.0160 (11)	0.0120 (11)	0.0112 (10)
C17	0.0747 (17)	0.0545 (13)	0.0479 (12)	−0.0054 (11)	−0.0024 (11)	0.0042 (10)
C18	0.0556 (14)	0.0586 (13)	0.0475 (12)	−0.0072 (10)	0.0049 (10)	0.0085 (10)
C19	0.122 (2)	0.0779 (17)	0.0474 (13)	0.0249 (16)	0.0189 (15)	0.0076 (12)

### Geometric parameters (Å, º)

**Table:**

F1—C4	1.352 (2)	C8—H8	0.9300
N1—C11	1.348 (2)	C9—C10	1.473 (3)
N1—N2	1.372 (2)	C10—C11	1.376 (2)
N1—C13	1.434 (2)	C11—C12	1.478 (3)
N2—N3	1.298 (2)	C12—H12A	0.9600
N3—C10	1.362 (3)	C12—H12B	0.9600
O1—C9	1.222 (2)	C12—H12C	0.9600
C1—C6	1.392 (3)	C13—C18	1.376 (3)
C1—C2	1.393 (3)	C13—C14	1.380 (3)
C1—C7	1.460 (3)	C14—C15	1.379 (3)
C2—C3	1.381 (3)	C14—H14	0.9300
C2—H2	0.9300	C15—C16	1.384 (3)
C3—C4	1.360 (3)	C15—H15	0.9300
C3—H3	0.9300	C16—C17	1.382 (3)
C4—C5	1.370 (3)	C16—C19	1.513 (3)
C5—C6	1.374 (3)	C17—C18	1.378 (3)
C5—H5	0.9300	C17—H17	0.9300
C6—H6	0.9300	C18—H18	0.9300
C7—C8	1.328 (3)	C19—H19A	0.9600
C7—H7	0.9300	C19—H19B	0.9600
C8—C9	1.471 (3)	C19—H19C	0.9600
			
C11—N1—N2	111.07 (15)	C11—C10—C9	128.12 (19)
C11—N1—C13	129.77 (16)	N1—C11—C10	103.79 (17)
N2—N1—C13	118.99 (15)	N1—C11—C12	124.33 (17)
N3—N2—N1	106.75 (15)	C10—C11—C12	131.83 (18)
N2—N3—C10	109.28 (15)	C11—C12—H12A	109.5
C6—C1—C2	117.65 (18)	C11—C12—H12B	109.5
C6—C1—C7	122.83 (18)	H12A—C12—H12B	109.5
C2—C1—C7	119.50 (19)	C11—C12—H12C	109.5
C3—C2—C1	121.6 (2)	H12A—C12—H12C	109.5
C3—C2—H2	119.2	H12B—C12—H12C	109.5
C1—C2—H2	119.2	C18—C13—C14	120.54 (18)
C4—C3—C2	118.14 (19)	C18—C13—N1	118.91 (17)
C4—C3—H3	120.9	C14—C13—N1	120.55 (18)
C2—C3—H3	120.9	C15—C14—C13	118.9 (2)
F1—C4—C3	119.19 (19)	C15—C14—H14	120.5
F1—C4—C5	118.0 (2)	C13—C14—H14	120.5
C3—C4—C5	122.79 (19)	C14—C15—C16	121.8 (2)
C4—C5—C6	118.5 (2)	C14—C15—H15	119.1
C4—C5—H5	120.7	C16—C15—H15	119.1
C6—C5—H5	120.7	C17—C16—C15	117.70 (19)
C5—C6—C1	121.32 (19)	C17—C16—C19	121.7 (2)
C5—C6—H6	119.3	C15—C16—C19	120.6 (2)
C1—C6—H6	119.3	C18—C17—C16	121.5 (2)
C8—C7—C1	128.0 (2)	C18—C17—H17	119.2
C8—C7—H7	116.0	C16—C17—H17	119.2
C1—C7—H7	116.0	C13—C18—C17	119.4 (2)
C7—C8—C9	121.4 (2)	C13—C18—H18	120.3
C7—C8—H8	119.3	C17—C18—H18	120.3
C9—C8—H8	119.3	C16—C19—H19A	109.5
O1—C9—C8	122.50 (19)	C16—C19—H19B	109.5
O1—C9—C10	120.09 (18)	H19A—C19—H19B	109.5
C8—C9—C10	117.41 (19)	C16—C19—H19C	109.5
N3—C10—C11	109.11 (17)	H19A—C19—H19C	109.5
N3—C10—C9	122.71 (17)	H19B—C19—H19C	109.5
			
C11—N1—N2—N3	0.2 (2)	C8—C9—C10—C11	−174.80 (18)
C13—N1—N2—N3	175.93 (15)	N2—N1—C11—C10	−0.2 (2)
N1—N2—N3—C10	−0.1 (2)	C13—N1—C11—C10	−175.33 (17)
C6—C1—C2—C3	−0.7 (3)	N2—N1—C11—C12	177.50 (18)
C7—C1—C2—C3	177.77 (18)	C13—N1—C11—C12	2.3 (3)
C1—C2—C3—C4	−0.5 (3)	N3—C10—C11—N1	0.1 (2)
C2—C3—C4—F1	−179.26 (18)	C9—C10—C11—N1	177.11 (18)
C2—C3—C4—C5	1.1 (3)	N3—C10—C11—C12	−177.3 (2)
F1—C4—C5—C6	−179.99 (18)	C9—C10—C11—C12	−0.3 (3)
C3—C4—C5—C6	−0.3 (3)	C11—N1—C13—C18	125.1 (2)
C4—C5—C6—C1	−1.0 (3)	N2—N1—C13—C18	−49.8 (2)
C2—C1—C6—C5	1.5 (3)	C11—N1—C13—C14	−54.7 (3)
C7—C1—C6—C5	−176.93 (19)	N2—N1—C13—C14	130.5 (2)
C6—C1—C7—C8	4.2 (3)	C18—C13—C14—C15	−1.6 (3)
C2—C1—C7—C8	−174.2 (2)	N1—C13—C14—C15	178.08 (18)
C1—C7—C8—C9	178.30 (18)	C13—C14—C15—C16	0.3 (3)
C7—C8—C9—O1	−1.0 (3)	C14—C15—C16—C17	1.6 (3)
C7—C8—C9—C10	178.42 (18)	C14—C15—C16—C19	−177.6 (2)
N2—N3—C10—C11	0.0 (2)	C15—C16—C17—C18	−2.3 (3)
N2—N3—C10—C9	−177.20 (17)	C19—C16—C17—C18	176.8 (2)
O1—C9—C10—N3	−178.71 (19)	C14—C13—C18—C17	0.9 (3)
C8—C9—C10—N3	1.9 (3)	N1—C13—C18—C17	−178.76 (18)
O1—C9—C10—C11	4.6 (3)	C16—C17—C18—C13	1.1 (3)

### Hydrogen-bond geometry (Å, º)

Cg1 is the centroid of the C13–C18 benzene

**Table:**

*D*—H···*A*	*D*—H	H···*A*	*D*···*A*	*D*—H···*A*
C12—H12*C*···N3^i^	0.96	2.49	3.399 (3)	158
C2—H2···*Cg*1^ii^	0.93	2.91	3.650 (2)	138

Symmetry codes: (i) *x*+1, *y*, *z*; (ii) *x*, *y*, *z*+1.
